# External Fixation for Unstable Proximal Humerus Fractures: A Narrative Review

**DOI:** 10.7759/cureus.105204

**Published:** 2026-03-14

**Authors:** Lyubomir Rusimov

**Affiliations:** 1 Trauma and Orthopaedics, University Multiprofile Hospital for Active Treatment With Emergency Medicine “N. I. Pirogov”, Sofia, BGR

**Keywords:** external fixation, minimally invasive fixation, percutaneous fixation, pin tract infection, proximal humerus fractures

## Abstract

Proximal humerus fractures (PHFs) are common injuries, particularly in the elderly population, and their optimal management remains controversial, especially in unstable fracture patterns. External fixation (EF) represents a minimally invasive treatment option that preserves the fracture hematoma and soft tissues while providing stable fixation. The aim of this narrative review is to summarize and critically appraise the available literature on EF as a treatment option for unstable PHFs. Biomechanical studies indicate that, with an optimized number and configuration of pins, the mechanical strength of EF approaches that of locking plate constructs. Despite heterogeneity in the EF systems used across clinical studies, the underlying principles remain similar. The technique is based on closed reduction and percutaneous fixation, resulting in minimal blood loss, shorter operative time, and a relatively short learning curve. These factors, together with the possibility of performing the procedure in the supine position, make EF particularly suitable for polytrauma patients and elderly individuals requiring minimally invasive surgery. Additional advantages include the ability to initiate early postoperative range of motion and removal of the external fixator in an outpatient setting without anesthesia. Clinical outcomes are generally favorable, particularly in two- and three-part PHFs, whereas results in four-part fractures are less predictable and associated with higher complication rates. The most frequently reported adverse event is pin tract infection, which typically does not require revision surgery and is usually managed successfully with systemic antibiotics. However, comparisons across studies are limited by heterogeneity in study design, fracture patterns, EF constructs, and outcome assessment methods. Further comparative and randomized studies are needed to better define the role of EF in the treatment algorithm of unstable PHFs.

## Introduction and background

Proximal humerus fractures (PHFs) account for approximately 5% of all fractures and represent 53% of shoulder girdle injuries [1,2]. Among patients older than 65 years, they rank as the third most common fracture, following distal radius and femoral neck fractures. In terms of overall incidence, they have been reported to rank around seventh among all fractures [1-3]. Although the majority of these fractures can be managed nonoperatively [4], the anticipated rise in their incidence is likely to lead to an increasing number of surgical interventions [2,5]. Various fixation techniques are currently available for the treatment of PHFs, including percutaneous pinning [6], intramedullary nailing [7], arthroplasty [8], and the current gold standard, locking plates [9], used with or without different augmentation strategies [10]. A less commonly reported approach to these fractures is external fixation (EF) [11]. Although EF is often perceived as unconventional or unnecessary in an era of increasingly advanced fixation technologies [11] - and is more commonly associated with revision surgery for infected PHFs [12] or deformity correction [13] - several authors have recently reported favorable outcomes with its use as a primary fixation method in unstable PHFs [14-16]. This renewed interest is related to potential biomechanical and clinical advantages, including limited soft-tissue disruption, shorter surgical time, stable fixation in osteoporotic bone, and a potentially reduced complication rate [16,17].

Therefore, the aim of this narrative review is to summarize and critically appraise the available literature on EF as a treatment option for unstable PHFs. To our knowledge, this is the first review specifically addressing this topic.

## Review

Materials and methods

For this narrative review, an exploratory literature search was conducted in the MEDLINE (via PubMed) and Scopus databases. The search strategy combined the terms “proximal humerus fractures” with “external fixation,” “external fixator,” or “ex fix.” The search was initially performed in February 2026 to capture the most recent available literature. No specific timeframe restriction was applied during the literature search in order to capture all available studies on this relatively limited topic. Only articles published in English were considered. As this work was designed as a narrative review, the search strategy aimed to identify relevant literature for qualitative synthesis rather than to perform a systematic review or meta-analysis. Retrieved studies were critically evaluated, and relevant clinical and biomechanical studies were included in the detailed review. The reference lists of selected articles were also screened to identify additional studies of interest.

Titles and abstracts of the retrieved articles were screened for relevance, followed by full-text evaluation of potentially eligible studies. Relevant data regarding study design, patient characteristics, fixation constructs, and reported outcomes were extracted and summarized for qualitative analysis. Due to the heterogeneity and limited number of available studies, a formal risk-of-bias assessment was not performed.

For biomechanical studies, inclusion criteria were cadaveric or synthetic humeri models of PHFs evaluating the biomechanical properties of external fixator constructs with quantitative stability outcomes. Studies were excluded if they involved animal models, constructs not involving EF, or insufficient biomechanical data to assess stability. 

The inclusion criteria for clinical studies were closed PHFs due to trauma in patients aged ≥18 years, a minimum of six patients per study, at least six months of follow-up, and treatment exclusively with EF. Exclusion criteria included studies on pathological fractures, open fractures, infected cases, or deformity correction procedures, patients younger than 18 years, studies with fewer than six patients, or follow-up shorter than six months.

Overall biomechanical results

Available biomechanical data on EF for PHFs demonstrate encouraging construct stability, which increases progressively with optimized wire number and configuration [18-20]. In their biomechanical study, Castoldi et al. evaluated the stability of various percutaneous wire configurations for two-part surgical neck fractures in synthetic humeri, with and without external fixator augmentation, and compared their performance with locking plate fixation under torsional, bending, and compressive loading. Biomechanical testing demonstrated that linking percutaneous wires with an external fixator markedly increased construct stiffness across torsion, bending, and compression. When four 3-mm wires were placed in a box configuration and externally linked, stability approached that of locking plate fixation without significant differences. In addition, constructs using 3-mm wires showed greater torsional stability than those using 2.5-mm wires [18].

In a paired cadaveric model with three-part fractures, Greinwald et al. found that a six-wire minimally invasive external fixator provided semi-rigid stability, with slightly greater lateral motion than plates but similar medial motion and head rotation, and no failures under physiologic loads [19]. Similarly, in cadaveric three-part fractures, Harbrecht et al. tested the Galaxy Fixation Shoulder System in two configurations: Construct A, with six wires, and Construct B, with eight wires crossing the fracture and aligned in a single plane. Construct B showed higher stability and less fracture-site motion than Construct A, approaching the performance of locking plates [17]. 

Together, these studies indicate that external fixator constructs with increasing wire number and optimized configuration progressively enhance biomechanical stability.

Overall clinical results

Across the available clinical literature, studies evaluating EF for unstable PHFs generally report favorable functional outcomes, with results consistently influenced by fracture complexity. In the largest published series, Blonna et al. reported outcomes in 188 patients with heterogeneous fracture patterns (two-, three-, and four-part fractures according to the Neer classification), achieving a mean Oxford Shoulder Score of 42.6 ± 0.5, a Subjective Shoulder Value of 85.5 ± 1.3, and low pain levels (visual analog scale (VAS) = 1 ± 0.13) [21]. Moro et al., in a cohort of 118 patients with AO type A, B, and C fractures, reported a mean Constant-Murley score of 75.5 ± 8.0 and a mean VAS pain score of 1.7 ± 2.08. Importantly, outcomes were excellent in simpler type A fractures and progressively lower, though still satisfactory, in types B and C fractures [22].

This trend is further supported by studies with more selected fracture patterns. Parlato et al. reported a mean Constant-Murley score of 84 points in 84 patients with two- and three-part fractures, along with good UCLA scores and minimal disability on the QuickDASH [23]. In highly selected cohorts limited to two-part fractures, such as the study by Benetos et al., excellent functional outcomes were observed across all scoring systems, with mean Constant scores exceeding 90 points at one-year follow-up [24].

Only two comparative studies evaluating EF in unstable PHFs were identified. Blonna et al. compared closed reduction and percutaneous pinning with EF in two-, three-, and four-part PHFs and reported superior functional outcomes and a lower complication rate in the EF group [25]. Vicenti et al. compared EF with locking plate fixation in patients with three- and four-part PHFs. When outcomes were stratified by age, patients younger than 65 years achieved higher Constant-Murley scores following plate fixation compared with EF (mean 79 ± 15 vs. 58 ± 4; p = 0.008). In contrast, among patients aged 65 years and older, no clinically relevant differences in Constant-Murley scores were observed at final follow-up between the two treatment groups (plate fixation: 69 ± 15; EF: 63 ± 7) [26]. Another comparative study, not comparing different fixation methods but rather configurations of the same EF technique, demonstrated that constructs using a higher number of wires (four pairs) were associated with better functional outcomes compared with constructs using fewer wires (three pairs) [27].

Except for fracture type, patient age, and number of wires, functional outcomes also appeared to be influenced by the type of fixator. Lower Constant-Murley and DASH scores were reported in a study using an Ilizarov ring fixator, as well as in a study employing a hybrid construct with a single half-ring configuration [22,28]. 

Beyond the generally favorable functional outcomes, several practical advantages of EF have been reported. Mean operative time is relatively short, ranging from approximately 22 ± 8.2 minutes for monoplanar constructs [16] to 66 ± 2.3 minutes in larger series [21] and 67 ± 10 minutes for circular (Ilizarov-type) fixators [28]. Intraoperative blood loss is described as minimal to nearly negligible, and the technique preserves the fracture hematoma due to its percutaneous nature [23]. Most authors advocate early postoperative mobilization with immediate or very early initiation of passive range of motion [15,21-31]. Additionally, fixator removal is typically performed in the outpatient setting without anesthesia, most commonly between five weeks postoperatively [22] and up to a maximum of approximately 12 weeks [29], depending on fracture healing and study protocol. A relatively short learning curve has also been suggested [15]. From an economic perspective, findings are conflicting: some authors consider EF a cost-effective option suitable for resource-limited settings [30], whereas others report higher overall costs compared with standard locking plate fixation [22], likely depending on the type of construct used.

Overall complications

Overall complication rates are generally low but vary among studies. In the largest series by Blonna et al. (188 patients), the overall complication rate was 27%, with pin tract infection (PTI) (8%), pin loosening (4.7%), and avascular necrosis (AVN) (4.3%) being the most common complications [21]. Despite this, the revision rate was only 3%. Notably, a limited deltopectoral approach was used in 68 patients with more complex fractures, which may have influenced the incidence of AVN [21].

Moro et al., in a cohort of 118 patients with AO type A-C fractures, reported 11% loss of reduction, along with isolated cases of superficial and deep infection, pin displacement, intra-articular pin penetration, and four cases of AVN [22]. In contrast, Scaravilli et al. (110 patients) and Parlato et al. (84 patients), both including predominantly two- and three-part fractures, reported no major complications [15,23].

Complication rates appear to increase with fracture complexity. Across studies, AVN, nonunion, and malunion are reported at relatively low rates, whereas pin tract infection is consistently the most frequently observed complication. In the small Ilizarov series by Meselhy et al. (14 patients), pin tract infection occurred in the majority of cases, suggesting a potential influence of fixator design [28].

Shabtai et al. specifically investigated the incidence and severity of infection in 46 patients with two-, three-, and four-part PHFs treated with closed reduction and EF [31]. Infection occurred in 18 patients (39.1%), of which 17 were superficial pin tract infections. The most commonly isolated organisms were gram-positive bacteria, particularly Staphylococcus epidermidis (10.8%) and Staphylococcus aureus (6.5%). Infection rates were strongly associated with the number of pins used: 81% of patients treated with five pins developed infection compared with 23% of those treated with four pins and none of those treated with three pins (p = 0.001). No association was found with age, concomitant injuries, or comorbidities. Both fracture complexity and the number of pins were identified as risk factors, although their interdependence makes it difficult to determine the primary contributor. Most infections were successfully managed with systemic antibiotics, and none required revision of the fixation construct [31]. 

Socio-psychological intolerance, a complication specific to EF, is infrequently reported. Because treatment success depends on patient compliance and acceptance, the external nature of the device may affect comfort and daily activities [19]. However, Blonna et al. reported no cases of intolerance, describing the fixator as lightweight, positioned close to the skin, and easily concealed under clothing [21,25]. Only Monga et al. reported one case of psychological intolerance, in which the patient considered the device socially unacceptable and required anxiolytic medication [30].

The overall clinical outcomes and complications are summarized in Table 1.

**Table 1:** Clinical outcomes and complications of external fixation for proximal humerus fractures. AO, Arbeitsgemeinschaft für Osteosynthesefragen; AVN, avascular necrosis; CMS, Constant-Murley score; JESS, Joshi external stabilization system; OSS, Oxford Shoulder score; PTI, pin tract infection; QuickDASH, Quick Disabilities of the Arm, Shoulder and Hand; SSV, subjective shoulder value; VAS, visual analog scale

Study (year)	n	Mean age (year)	Fracture type	Fixator type	Functional outcomes (final follow-up)	Operative time (min)	Complications	Revision
Martin et al. (2006) [32]	62	70	Neer 2-3	Monoplanar (Hoffmann II)	CMS 84	45	1 PTI, 1 redisplacement	2 cases
Ebraheim et al. (2007) [29]	64	55	Neer 2-3	Mini external fixator	Neer score: 63% excellent	60 (35-90)	2 nonunion, 2 deep inf., 4 displacement	No
Monga et al. (2009) [30]	20	38	Neer 2-4	AO external fixator	Neer score 85	-	7 PTI, 1 nonunion, 1 psych. intolerance	1 case
Blonna et al. (2010) [25]	188	~67	Neer 2-4	Hybrid	OSS 42.6; SSV 85.5; VAS 1.0	66 ± 2.3	PTI 8%, AVN 4.3%, pin loosening 4.7%	3%
Benetos et al. (2012) [24]	18	39	Neer 2	Monoplanar (TGF)	CMS 93; OSS 43.2; QuickDASH 5.3	45	1 PTI	No
Gupta et al. (2012) [33]	16	57.5	Neer 2-3	JESS	CMS 78.1 ± 9.6; VAS 2.1	-	1 PTI, 1 pin loosening	No
Zhang et al. (2012) [34]	32	56	Neer 2-4	Mini fixator	Neer score 83.2 ± 12.5	29 ± 12	1 collapse, 2 pin loosening	3 cases
Shabtai et al. (2013) [31]	46	50	Neer 2-4	Hoffmann II	Infection study	-	PTI 39.1%	No
Parlato et al. (2014) [23]	84	61	Neer 2-3	TGF (Gex-Fix)	CMS 84; OSS 41.2; QuickDASH 5.5	-	None	No
D’Ambrosi et al. (2016) [35]	32	66.8	Neer 3-4	Galaxy	OSS 88.9 ± 8.7; QuickDASH 3.1	-	PTI 9.4%, 1 malunion	No
Meselhy et al. (2017) [28]	14	42.9	Neer 2-3	Ilizarov	CMS 73.1; DASH 31.8; VAS 3.2	67 ± 10	PTI, 1 AVN	No
Gumina et al. (2019) [27]	52	63.1	Neer 3	Galaxy	Relative CMS 93% vs 86%	-	2 PTI, 1 nonunion	No
Moro et al. (2019) [22]	118	67.7	AO A-C	Hybrid (FraMo)	CMS 75.5 ± 8.0; VAS 1.71 ± 2.08	41 ± 10	11% loss red., 4 AVN	3 cases
Vicenti et al. (2019) [26]	24	62	Neer 3-4	External fixator	CMS 63 ± 7 (≥65 years)	-	1 PTI, 1 loss red.	No
Maluta et al. (2020) [16]	17	69.7	Neer 3-4	Dedicated EF	CMS 85 ± 9.9 (12 mo)	22 ± 8.2	2 PTI, 1 clamp loosening	No
Scaravilli et al. (2022) [15]	110	65.2	Neer 2-3	DOS system	DASH ↓ 75→29; OSS ↓ 47→28	-	None major	No
Thambusamy et al. (2023) [36]	10	-	Neer 2-4	JESS (6-pin)	CMS 60% excellent, 40% good	-	PTI 10%, malunion 10%	No
Sinha et al. (2025) [14]	28	65.2	Neer 2-4	JESS	CMS 75% good, 25% excellent	-	PTI, loosening (28.6%)	No

Surgical technique 

Fracture Reduction Techniques

Fracture reduction is generally performed under general anesthesia, with patients positioned either supine or in the beach-chair/semi-beach-chair position [15,21-34]. Supine positioning is frequently reported across several series and is particularly advantageous in polytrauma patients, allowing safe anesthesia management and unobstructed fluoroscopic control [21-24,28,29,34].

Several authors defined acceptable or anatomical reduction using similar radiographic criteria, most commonly residual displacement of less than 1 cm and angulation below 45° [24,29,33]. Some studies applied stricter thresholds; Zhang et al. defined functional reduction as achieving more than 75% cortical contact between major fragments, residual angulation below 20°, and preservation of the inferomedial calcar without a defect [34].

In the reviewed literature, fracture reduction was predominantly achieved using closed techniques based on ligamentotaxis, arm positioning, and controlled manipulation [15,16,21-23,26,29,32,33]. Reduction was facilitated by applying longitudinal traction and rotational forces to the arm, allowing indirect manipulation of fracture fragments and restoration of alignment [15,29,32].

Initial reduction was frequently performed with the shoulder abducted between 40° and 60° and the elbow flexed at approximately 70°-90°, while longitudinal traction was applied through the flexed elbow [29,34]. Correction of anterior or posterior angulation was achieved by elevating or lowering the arm, combined with counterpressure applied at the apex of the deformity [29,34]. Residual displacement or angulation was addressed by repeating these maneuvers under fluoroscopic control [29,34]. 

Several studies described a stepwise reduction sequence based on progressive abduction. After initial positioning, the arm was abducted to 90° and gradually increased to 120°-130°, allowing engagement of the proximal fragment beneath the acromion to act as a fulcrum for reduction [21]. Diaphyseal retropulsion and controlled counterpressure were then applied to overcome muscular forces and complete alignment [21,25,26].

When closed manipulation alone was insufficient, percutaneous reduction techniques were employed. Temporary Kirschner wires or pins were introduced into the humeral head or shaft and used as joysticks to assist fragment control [21,23-25]. Reduction of the greater tuberosity, when displaced, was achieved using threaded wires or percutaneous Kirschner wires acting as joysticks to grasp or tension the rotator cuff tendons and restore anatomical position [27]. Reduction of the lesser tuberosity, although considered of lesser functional importance, was similarly achieved using percutaneous joystick-assisted maneuvers and stabilized under fluoroscopic guidance [22].

In the largest available clinical series by Blonna et al., closed reduction was primarily indicated for displaced type A fractures and for selected type B and C fractures without significant tuberosity displacement. When satisfactory alignment could not be achieved using closed or percutaneous techniques, a limited open reduction through a small deltopectoral or transdeltoid incision was selectively performed to obtain acceptable reduction [21,25].

Fixation Construct and Principles of Fixation

The primary principle of EF in PHFs is to obtain stable fixation by transferring load from the weak cancellous bone of the humeral head to the stronger lateral cortical bone, thereby reducing the risk of secondary displacement [21]. The earliest description of EF for PHFs was provided by Kristiansen, who demonstrated that stable fixation could be achieved with minimal soft-tissue dissection using a deltopectoral approach and percutaneous pin placement into the humeral head and shaft [37]. In this original technique, two half-pins were typically inserted into the humeral head, with additional pins or sutures used to control displaced tuberosities when required. Shaft pins were introduced from the lateral cortex and advanced to just penetrate the medial cortex [37].

In subsequent studies, fixation was consistently performed after closed fracture reduction and under fluoroscopic guidance to control pin trajectory, depth relative to the articular surface, and overall construct alignment [21,22,25,29,32,37]. Monolateral EF systems were widely adopted, with variations in pin diameter, number, and orientation. Martin et al. used the Hoffmann II EF system, placing two parallel 5-mm screws into the upper lateral humeral head, oriented in the transverse plane and advanced toward the subchondral bone without penetrating the articular surface [32]. Two additional screws were placed in the humeral diaphysis, after which fracture reduction and fixation were completed by assembling the external fixator. In fracture-dislocation patterns, these screws also served as joysticks to restore the humeral head to its anatomical position [32].

Ebraheim et al. applied a mini external fixator using four 2.5-mm terminally threaded pins, with two pins inserted into the humeral head and two into the shaft, providing subchondral support while avoiding joint penetration [29]. Monga et al. reported the use of a standard AO external fixator based on similar principles of lateral cortical purchase and head-shaft stabilization [30].

More complex multiplanar constructs were described using Joshi-derived EF systems. Gupta et al. employed the Joshi external stabilization system (JESS), placing three pins in the humeral head with approximately 30° divergence and three additional pins in the proximal shaft in a similar configuration, supplemented by a distal diaphyseal pin; all pins were interconnected by rods and clamps to form a rigid multiplanar frame [30]. Sinha et al. and Thambusamy et al. reported comparable JESS constructs using multiple Kirschner wires placed in the tuberosity, calcar, and neck regions, arranged to create a tension-based stabilization system adapted to fracture morphology and bone quality [14,36].

Tension-based fixation systems were described by Parlato et al. and Benetos et al. using the Tension Guide Fixator [23,24]. In these constructs, two parallel Kirschner wires were introduced from the upper lateral aspect of the humeral head and advanced into the medullary canal. After reduction, two threaded half-pins were placed in the humeral diaphysis, and the Kirschner wires were tensioned and connected to an “L”-shaped body to create a stable tension construct. In three-part fractures, the joystick wire was incorporated into the final construct, whereas in two-part fractures, it was often removed after reduction [23,24].

Dedicated proximal humerus EF systems were introduced in later studies [21,27,35]. Blonna et al. and Gumina et al. used the Galaxy Fixation System, characterized by fully threaded 2.5-mm pins with long threaded segments designed to achieve purchase in both the lateral cortex and subchondral bone [21,27]. Pins were inserted from lateral to medial with divergent orientations, either crossing the fracture line (“pins-crossing-fracture” technique) or bridging the fracture with separate proximal and distal pin groups (“pins-bridging-fracture” technique). Additional pins were used in more complex fracture patterns or to stabilize displaced tuberosities [21].

Circular and hybrid fixation systems were reported in selected series. Meselhy et al. described an Ilizarov-based construct in which multiple half-pins were inserted into the humeral head from lateral, anterolateral, and posterolateral directions, stopping approximately 1 cm short of the articular surface, followed by placement of a distal arch below the deltoid insertion and final connection of both arches [28]. Moro et al. described a hybrid Ilizarov-derived system (FraMo), based on diaphyseal threaded pins acting as an anchoring point and a proximal half-ring through which multiple 2-mm threaded Kirschner wires were inserted in a trapezoidal orientation to stabilize the epiphyseal fragments [22].

Other technical variations were reported across the included studies. Zhang et al. described the use of widely spaced, intercrossing threaded pins within the humeral head, combined with additional diaphyseal pins, to enhance overall construct stability and resistance to rotational and varus collapse [34]. Vicenti et al. used four 2.5-mm terminally threaded Schanz pins placed from anterolateral to posteromedial directions, crossing the fracture with divergent orientation and reaching the subchondral bone [26]. Maluta et al. described a six-Kirschner wire construct with predefined entry points and calcar support, stabilized with bars and clamps [16]. Scaravilli et al. reported a modular system combining crossed Kirschner wires with diaphyseal pins connected by a telescopic carbon bar, allowing fine postoperative adjustment of compression or distraction after fixation [15].

Tuberosity fixation was addressed separately in several series [22,27]. Gumina et al. described fixation of the greater tuberosity using threaded wires introduced in craniocaudal and lateromedial directions, often transfixing the medial cortex to improve stability [22,27]. Additionally, Martin et al. used an upper anterior pin for fixation of the lesser tuberosity fragment when the fragment was sufficiently large [32].

Pitfalls, Prevention, and Technical Considerations

Careful attention to pin placement is essential to avoid neurovascular injury. During insertion of proximal and greater tuberosity pins, the axillary nerve and posterior humeral circumflex artery are at risk; external rotation of the shoulder during pin placement was consistently recommended to displace these structures away from the humeral neck [29,33]. Radial nerve injury was avoided by limiting distal pin placement proximal to the deltoid tuberosity and preventing posterior advancement of pins [29,32,33]. Anterior pin placement carries a risk to the cephalic vein, musculocutaneous nerve, biceps tendon, and anterior branch of the axillary nerve; therefore, careful identification of anterior entry points and, when necessary, the use of small incisions with minimal blunt dissection were emphasized to reduce iatrogenic injury [29,33,35].

Pin overpenetration represents an additional technical hazard. Across studies, pins were intentionally stopped short of the articular surface - typically between 3 and 10 mm beneath the subchondral bone - to balance fixation strength with avoidance of cartilage injury. Overpenetration of the medial cortex during greater tuberosity pin insertion was specifically cautioned against, because of the proximity of neurovascular structures [21,25,27-29,34].

Several authors highlighted the importance of testing reduction maneuvers before definitive pin insertion and maintaining the limb in a stable, controlled position during wire placement to prevent loss of reduction and pin malposition [22,35]. Slow pin insertion with frequent fluoroscopic checks was advised to confirm pin trajectory and depth and to avoid overpenetration of the medial cortex or articular surface [29,35].

Pin diameter, configuration, and construct stability were also identified as critical factors. Placement of multiple pins in different planes was reported to improve rotational stability, particularly in osteoporotic bone, while partially or fully threaded pins were favored to increase pullout strength and reduce the risk of loosening [22,29,33]. In cases of severe comminution or poor bone quality, fixation was augmented by increasing the number of wires or temporarily retaining reduction pins within the final construct to enhance overall stability [22,30]. Additionally, larger diameter Steinmann pins (5 mm) limit the available space for placement of multiple pins compared with smaller-diameter pins (2.5-3 mm) [36].

Technique-specific issues were reported with certain constructs. Blonna et al. observed that pin backing out and iatrogenic lateral cortical fractures occurred exclusively with the pins-crossing-fracture technique, emphasizing the importance of appropriate pin orientation and adequate lateral cortical support [21]. Avoidance of excessive stress concentration at pin entry points was highlighted as a key preventive measure [21,27]. Furthermore, fractures with loss of lateral cortical integrity were excluded, as an intact lateral cortex is essential for stable pin insertion; when the proximal lateral cortex is compromised, locking plate fixation was generally indicated [21,25].

Postoperative pin-site care was consistently emphasized to reduce the risk of pin tract infection. Reported protocols included daily or twice-daily cleaning with hydrogen peroxide, alcohol, chlorhexidine, or povidone-iodine solutions, with dressing changes performed either daily or weekly depending on institutional practice [16,26,27,29,33,34]. Early initiation of pin care from the first postoperative day was commonly recommended [29,33].

## Conclusions

Biomechanical evidence suggests that, with an optimized number and configuration of pins, the stability of EF can approach that of locking plate constructs. Despite heterogeneity in the EF systems used across clinical studies, the underlying principles remain consistent. The technique is based on closed reduction and percutaneous fixation, resulting in minimal blood loss, shorter operative time, and a relatively short learning curve. These factors, together with the ability to perform the procedure in the supine position, make EF particularly suitable for polytrauma patients and elderly individuals requiring minimally invasive surgery. Additional advantages include early initiation of postoperative range of motion and removal of the external fixator in an outpatient setting without anesthesia. Clinical outcomes are generally good and more favorable in two- and three-part PHFs, whereas results in four-part fractures are less predictable and associated with higher complication rates. Although reported complication rates vary among studies, revision rates remain low. The most frequently reported adverse event is pin tract infection, which rarely necessitates revision surgery and is usually managed successfully with systemic antibiotics.

However, interpretation of the available evidence is limited by heterogeneity in study design, fracture patterns, types of external fixators, and outcome assessment methods. Further high-quality comparative and randomized studies are needed to better define the role of EF in the management of unstable PHFs.
